# Osteochondral tissue coculture: An in vitro and in silico approach

**DOI:** 10.1002/bit.27127

**Published:** 2019-07-31

**Authors:** Ruikang Xue, Benedict Chung, Maryam Tamaddon, James Carr, Chaozong Liu, Sarah Harriet Cartmell

**Affiliations:** ^1^ School of Materials, Faculty of Science and Engineering University of Manchester Manchester UK; ^2^ Institute of Orthopaedics and Musculo‐Skeletal Science University College London London UK; ^3^ Manchester Imaging Facility University of Manchester Manchester UK

**Keywords:** additive tissue engineering, bilayered scaffold, coculture, finite element analysis, osteochondral tissue engineering

## Abstract

Osteochondral tissue engineering aims to regenerate functional tissue‐mimicking physiological properties of injured cartilage and its subchondral bone. Given the distinct structural and biochemical difference between bone and cartilage, bilayered scaffolds, and bioreactors are commonly employed. We present an osteochondral culture system which cocultured ATDC5 and MC3T3‐E1 cells on an additive manufactured bilayered scaffold in a dual‐chamber perfusion bioreactor. Also, finite element models (FEM) based on the microcomputed tomography image of the manufactured scaffold as well as on the computer‐aided design (CAD) were constructed; the microenvironment inside the two FEM was studied and compared. In vitro results showed that the coculture system supported osteochondral tissue growth in terms of cell viability, proliferation, distribution, and attachment. In silico results showed that the CAD and the actual manufactured scaffold had significant differences in the flow velocity, differentiation media mixing in the bioreactor and fluid‐induced shear stress experienced by the cells. This system was shown to have the desired microenvironment for osteochondral tissue engineering and it can potentially be used as an inexpensive tool for testing newly developed pharmaceutical products for osteochondral defects.

## Graphical abstract

An osteochondral tissue coculture system combining a dual‐chamber perfusion bioreactor and a bilayered scaffold is described. The authors have studied the system through both in vitro coculture of cartilage and bone cells, and in silico computational modelling of the microenvironment inside a µCT scanned scaffold during perfusion. This system was shown to have desired microenvironment for osteochondral tissue engineering and it can potentially be used as an inexpensive tool for testing newly developed pharmaceutical products for osteochondral defects.

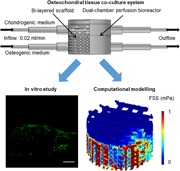

## 1 INTRODUCTION

Osteoarthritis of the synovial joint is a common cause of osteochondral defects. Osteoarthritis of the knee accounts for 83% of total osteoarthritis burden and affects around 250 million people globally (Vos et al., 2012). Injured cartilage does not heal spontaneously due to limited access to progenitor cells and scarce blood supply (Redman, Oldfield, & Archer, 2005).

Osteochondral tissue engineering aims to restore tissue that is functionally and mechanically comparable to native hyaline cartilage and its subchondral bone (Nukavarapu & Dorcemus, 2013). Given the distinct difference in structure and microenvironment of the two tissue types, osteochondral tissue engineers often employ bilayered scaffolds and bioreactors to provide different microenvironments to bone and cartilage layers and to facilitate nutrient and waste transport. Previously, our group cocultured chondrocytes and osteoblasts on a hyaluronate/β‐tricalcium phosphate (β‐TCP) bilayered scaffold in a dual‐chamber perfusion bioreactor for 7 days (Kuiper, Wang, & Cartmell, 2014). It demonstrated that the bioreactor was able to maintain the respective osteoblast and chondrocyte phenotype in each layer. However, lower mechanical strength and permeability of the scaffold were expected, as its chondral and osseous layers were manufactured independently before joining (Mano & Reis, 2007).

One way to improve scaffold mechanical stability is to produce a gradient structure through additive manufacturing techniques (Giannitelli, Accoto, Trombetta, & Rainer, 2014; Yousefi, Hoque, Prasad, & Uth, 2015). Additive manufacturing is a scalable process that can create complex and tuneable scaffolds from CAD models. It has been shown that the discrepancy between the CAD and the actual manufactured geometry can cause a significant change in the microenvironment inside a bioreactor through finite element analysis (FEA) (Hendrikson, van Blitterswijk, Verdonschot, Moroni, & Rouwkema, 2014). By combining microcomputed tomography (μCT) and FEA, the culture microenvironment of an actual manufactured scaffold can be studied.

Various immortalized cell lines have widely been used for osteochondral tissue engineering because they exhibit specific cell behavior observed in primary chondrocytes or osteoblasts with low cost and ease of use. Murine osteoblastic MC3T3‐E1 cells exhibit an osteoblast‐like developmental sequence, from proliferation to mineral deposition in vitro (Quarles, Yohay, Lever, Caton, & Wenstrup, 1992; Wang et al., 1999). For cartilage tissue, ATDC5 cells are often used as an in vitro model for skeletal development as they show a sequential chondrocyte differentiation process (Newton et al., 2012; Yao & Wang, 2013).

In this study, we aimed to describe an in vitro osteochondral perfusion coculture system—a novel additive manufactured bilayered scaffold inside a coculture bioreactor. The new bilayered scaffolds were designed to have improved integrity and permeability compared to the previous scaffolds. The coculture system was investigated in vitro through coculturing ATDC5 and MC3T3‐E1 cells on the respective chondral and osseous layers of the scaffold, as well as in silico through FEA of the microenvironment inside the scaffold (i.e., flow velocity, fluid‐induced shear stress, and differentiation media mixing) during the perfusion. The microenvironment inside the actual manufactured scaffold from µCT was compared with the CAD, and the effective microenvironment for osteochondral tissue engineering was discussed.

## 2 MATERIALS AND METHODS

### 2.1 Materials

Dulbecco's phosphate‐buffered saline (DPBS), minimum essential medium‐α modification (α‐MEM), Dulbecco's modified Eagle's medium/nutrient mixture F‐12 (DMEM/F12), fetal bovine serum (FBS), antibiotic antimycotic solution (A/B), l‐glutamine, β‐glycerophosphate (β‐GP), ascorbic acid, glutaraldehyde, hexamethyldisilazane (HMDS), and bovine Achilles tendon collagen were purchased from Sigma‐Aldrich. Insulin–transferrin–selenium (ITS) premix was purchased from Corning. Resazurin assay was purchased from Biolegend. LIVE/DEAD Cell Imaging Kit, Vybrant DiO Cell‐Labeling Solution (DiO), and Vybrant DiD Cell‐Labeling Solution (DiD) were purchased from Invitrogen.

### 2.2 Cell line and culture media

Mouse chondrogenic cell line ATDC5 and mouse osteoblastic cell line MC3T3‐E1 were purchased from Public Health England. ATDC5 and MC3T3‐E1 cells were maintained in the respective cartilage and bone growth media. The cartilage growth medium was composed of DMEM/F12 with 5% FBS and 1% A/B; and the bone growth medium was composed of α‐MEM with 10% FBS, 2 mM l‐glutamine and 1% A/B.

Chondrogenic and osteogenic media were prepared. More exactly, for the chondrogenic medium, the cartilage growth medium was supplemented with 0.2% ITS premix and 50 µg/ml ascorbic acid. For the osteogenic medium, the bone growth medium was supplemented with 10 mM β‐GP and 50 µg/ml ascorbic acid.

### 2.3 Scaffold fabrication

**Figure 1:**
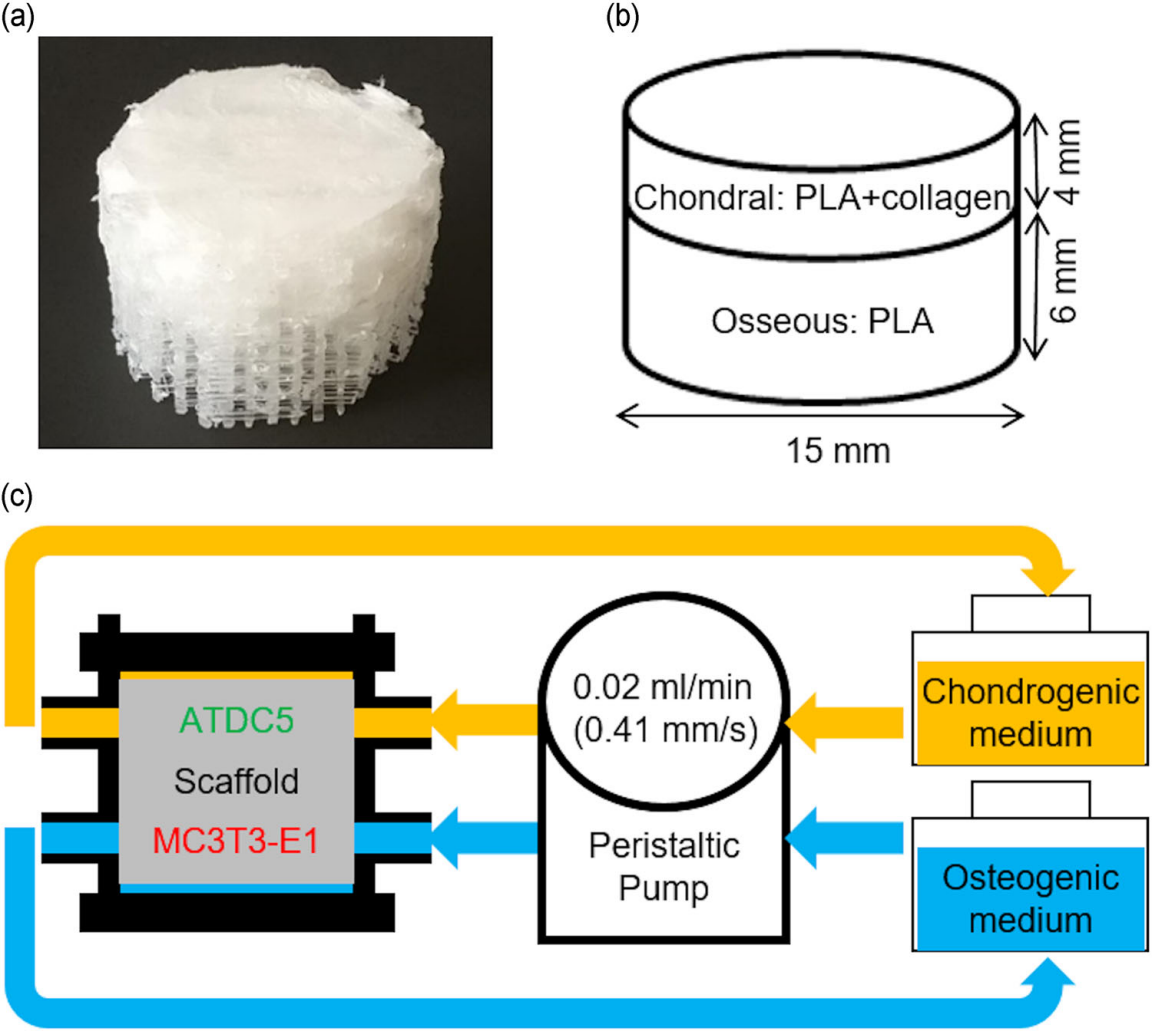
(a) Photograph of the bilayered scaffold. (b) Schematic of scaffold composition and geometry. (c) Schematic of perfusion coculture [Color figure can be viewed at wileyonlinelibrary.com]

Bilayered polylactic acid (PLA) scaffolds were used in this study (Figure 1a). The top osseous layer was composed of a coarse mesh of PLA struts with 1000 µm strut diameter and 1000–1500 µm strut spacing and infiltrated with type I collagen. The bottom chondral layer of the scaffold was composed of a fine mesh of PLA struts with 500 µm strut diameter and 500 µm strut spacing. The top and the bottom layers were separated with two layers of close‐packed struts with 500 µm strut diameter.

An additive manufacturing system (FlashForge Creater Pro) was used to fabricate PLA scaffolds with a 0.4 mm standard nozzle from the CAD; each scaffold was manufactured with one continuous print. Verbatim PLA filaments (1.75 mm, natural) were extruded at a nozzle temperature of 210°C, with a nozzle travel speed of 20 mm/s on the platform with a temperature of 35°C, and a layer height of 500 µm. The obtained PLA structures were then treated in a UV/Ozone reactor (Bioforce Nanosciences) for 5 min on each side to improve the surface wettability. The collagen suspension was produced according to a previously established method (Liu, Shen, & Han, 2011; Tamaddon, Walton, Brand, & Czernuszka, 2013). Briefly, a dispersion of 1% bovine Achilles tendon collagen in 0.05 M acetic acid solution (pH = 3.2) was homogenized on ice and degassed using centrifugation. The bi‐layered scaffold was then produced by casting the collagen dispersion into custom‐made 3D printed cylindrical resin molds (15 mm diameter, 10 mm height, Figure 1b), freezing them overnight at  −20°C and freeze‐drying them for 24 hr (Christ Alpha 1–2).

### 2.4 Cell seeding

The scaffolds were sterilized using 70% ethanol three times for 15 min, washed twice with DPBS for 5 min and were stored in α‐MEM in a humidified incubator at 37°C before use.

Before cell seeding, the scaffolds were dabbed with sterile tissue to remove excess liquid. A total of 500,000 ATDC5 cells in 100 µl chondrogenic medium were placed onto the top of the scaffold, followed by 2 hr incubation at 37°C. The scaffolds were then inverted and 500,000 MC3T3‐E1 cells in 200 µl osteogenic medium were placed onto the bottom of the scaffold, followed by 2 hr incubation at 37°C.

### 2.5 Perfusion coculture

The dual‐chamber perfusion bioreactor used in the study was described previously (Kuiper et al., 2014). Briefly, the cell‐seeded scaffold was put into the bioreactor. 30 ml chondrogenic medium and 30 ml osteogenic medium was added to the reservoirs connected to the respective top and bottom part of the bioreactor. Each bioreactor and the two reservoirs were then connected to a peristaltic pump equipped with 1.02 mm tubing (U205, Watson Marlow). Chondrogenic and osteogenic media were perfused through the respective top and bottom part of the coculture bioreactor at 0.5 rpm (~0.02 ml/min or 0.41 mm/s; Figure 1c). The differentiation media were changed every 3 days.

The scaffolds were harvested after 7 days of perfusion culture. Each scaffold was cut into two equal half‐cylinders with a surgical blade (Swann‐Morton) and placed in α‐MEM before immediate analysis. Each half‐cylindrical scaffold was considered as one sample.

### 2.6 Live/dead assay

To make the working solution, equal volumes of the LIVE/DEAD Cell Imaging Kit assay solution and cell culture medium were mixed. Samples were washed and then incubated in the working solution for 15 min at the room temperature. Fluorescence micrographs were taken at 488/515 and 570/602 nm excitation/emission wavelength for the respective viable and dead cells with a confocal laser scanning microscope (CLSM, Leica).

### 2.7 Resazurin assay

The resazurin assay was used to quantify the cell metabolic activity and proliferation in the whole scaffold, including both the bone and the cartilage section. Samples were washed with DPBS. After washing, each sample was incubated in 4 ml α‐MEM containing 10% resazurin assay solution at 37°C for 4 hr. Next, 100 µl culture medium was collected and its fluorescence intensity at excitation/emission 560/590 nm was measured with a Microplate Reader (BMG Labtech).

### 2.8 DiO and DiD cell tracking

DiO and DiD dyes were used to label the respective ATDC5 and MC3T3‐E1 cells before cell seeding to study the cell distribution inside the scaffold. The cell‐labeling solution was diluted in α‐MEM at 5:1,000 ratio to make the working solution. 500 µl working solution was added to the T25 cell culture flask and incubated for 30 min at 37°C. DiO‐ and DiD‐labeled cells were then seeded onto the scaffold as described previously. The harvested sample was evenly divided into three sections, namely, top, middle, and the bottom section (Figure 5a). Fluorescence micrographs were taken at 484/501 and 644/665 nm excitation/emission wavelength for the respective DiD and DiO label with a CLSM. MC3T3‐E1 and ATDC5 cell number at each section was counted with ImageJ (NIH).

### 2.9 Scanning electron microscopy

Samples were fixed with 1.5% glutaraldehyde solution at 4°C for 30 min, followed by dehydrating through ascending grades of ethanol (from 50% to 100%). Dehydrated samples were further dried by evaporation of the HMDS. Next, samples were mounted onto aluminum pin stubs (Agar Scientific) with Adhesive Carbon Tabs (Agar Scientific). Samples were sputter‐coated with Au/Pd before imaging with Phenom Pro desktop SEM (Phenom‐World) at approximately 500× magnification.

### 2.10 Finite element analysis

**Figure 2:**
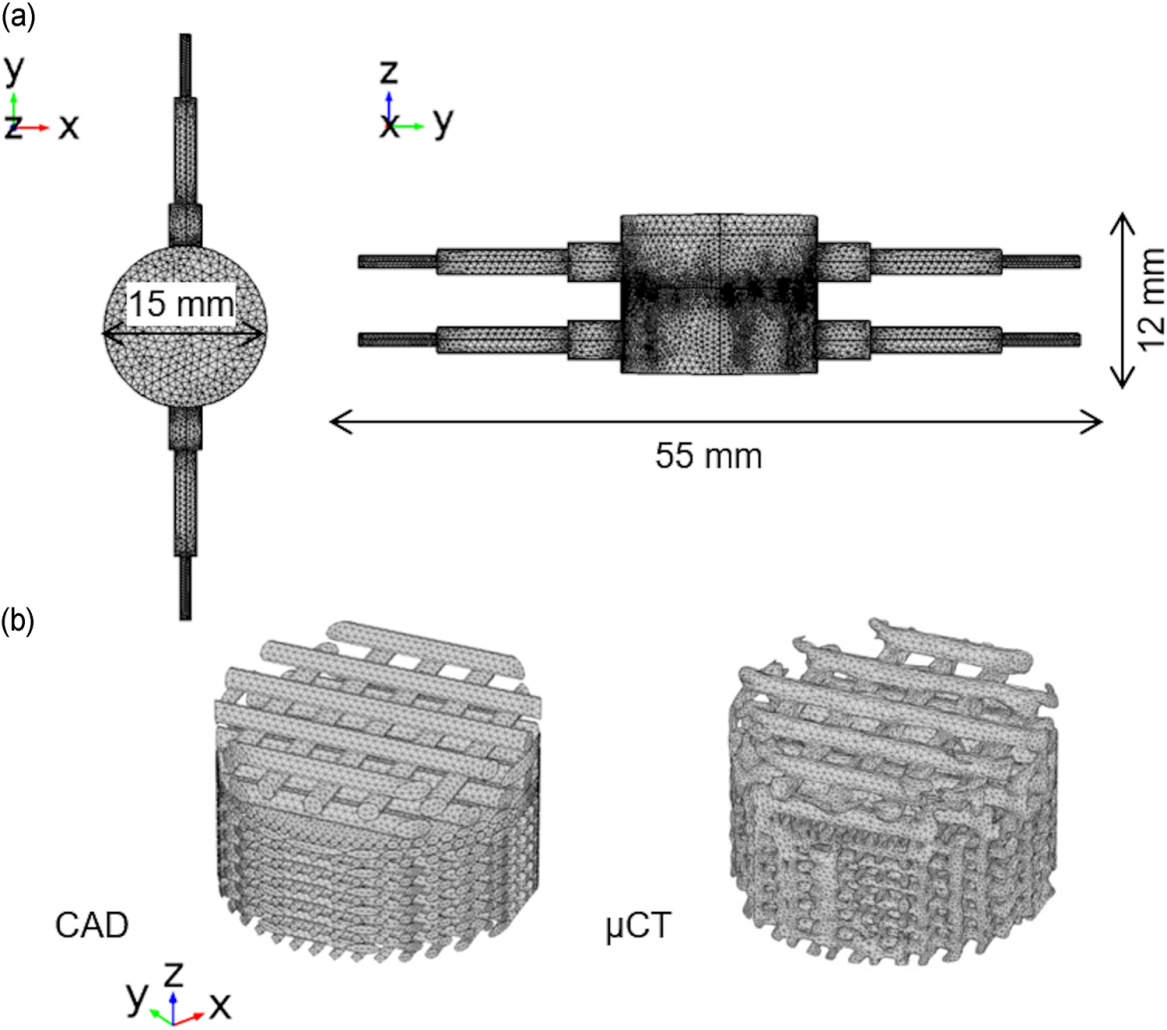
(a) Volume mesh of bioreactor chamber. (b) Volume mesh of scaffold geometry obtained from CAD or μCT, the collagen layer is not shown. CAD, computer‐aided design; μCT, microcomputed tomography [Color figure can be viewed at wileyonlinelibrary.com]

The chamber geometry (Figure 2a) of the coculture bioreactor was generated in COMSOL Multiphysics (COMSOL). To model the PLA scaffold, its geometry was obtained from either the CAD or the μCT scan of the actual manufactured scaffold.

For the model of the CAD scaffold, the CAD file was imported to COMSOL multiphysics and physics‐controlled normal mesh with boundary layers disabled was used to mesh the geometry (Figure 2b). A total of 468234 tetrahedral elements were generated with size ranging from 0.411 to 2.74 mm. For the model of the actual manufactured scaffold, scaffolds were μCT‐scanned using a Nikon XT H225 at 80 kV and 125 μA. A total of 3142 projections were captured with a 2000 × 2000 pixel detector, leading to a voxel size of 7.9 μm. μCT data were reconstructed with CT Pro 3D (Nikon) with beam hardening and center of rotation automatically calculated. Reconstructed data then was smoothed with bilateral filter and segmented with automatic thresholding in Avizo (FEI). As collagen had a very low attenuation under X‐ray illumination, the geometry from PLA was reconstructed. The finite element volume mesh was generated in a specialized meshing software Simpleware (Synopsys). More precisely, segmented μCT data of the scaffold generated from Avizo and the chamber geometry generated from COMSOL Multiphysics were imported to Simpleware. The scaffold and the chamber were aligned and a volume mesh with 1502281 tetrahedral elements was generated. The generated meshes were then imported to COMSOL Multiphysics (Figure 2b).

**Figure 3:**
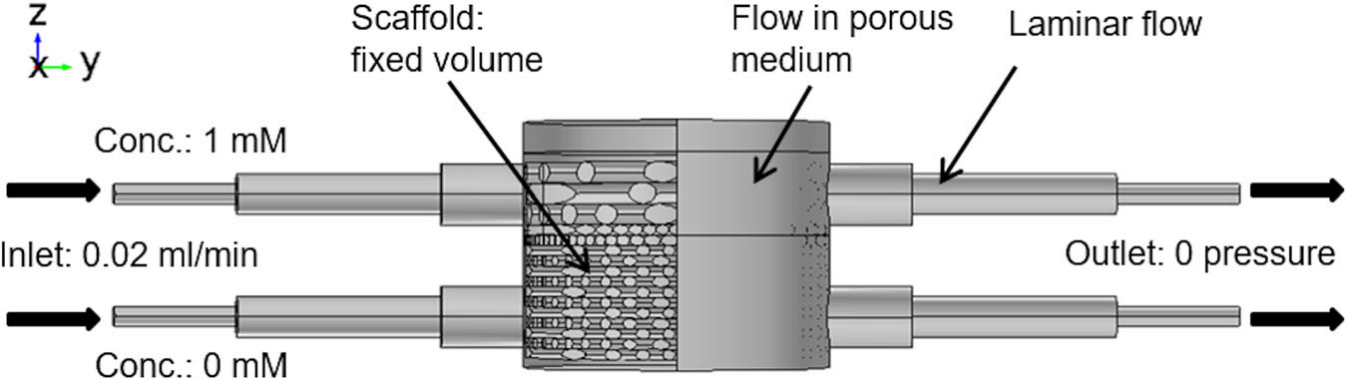
Boundary conditions used in the finite element model [Color figure can be viewed at wileyonlinelibrary.com]

In COMSOL multiphysics, the material properties of the perfusion media, namely, the dynamic viscosity of 1 × 10^−3^ Pa·s, the density of 1000 kg/m^3^, and diffusion coefficient of 2.907 × 10^−9^ m^2^/s (Holz, Heil, & Sacco, 2000) were used in the model. The single‐phase laminar flow module was used to calculate the flow velocity based on the Navier–Stokes equation. The flow field inside the collagen hydrogel in the chondral layer was modeled as flow in porous media using the Brinkman equation where 1 × 10^–12^ m^2^ permeability and 90% porosity were applied (Moreno‐Arotzena, Meier, Del Amo, & García‐Aznar, 2015). When coupled with the solid mechanics' module, the fluid‐induced shear stress (FSS) on the PLA struts was calculated; and when coupled with the transport of diluted species module, the concentration of diluted species was calculated. In the single‐phase laminar flow module, 0.02 ml/min laminar inflow was applied at the bioreactor inlets, 0 Pa pressure was applied at the bioreactor outlets and nonslip condition was applied on the bioreactor and scaffold wall (Figure 3). Assuming that the scaffold does not move during the perfusion, in the solid mechanics' module, a fixed volume constraint was applied to the scaffold. In the transport of diluted species module, 1 (for chondrogenic medium) and 0 mM (for osteogenic medium) concentrations were applied at the respective top and bottom inlets to study the differentiation media mixing. A stationary study step was created and results including flow velocity inside the chamber, FSS on the scaffold and differentiation media mixing, were obtained.

Models derived from the CAD image with different angles of scaffold rotation in the chamber was also studied. Here, 30° angle of rotation (angle between the PLA strut on the top layer of the scaffold and y axis) was used due to the ease of meshing of the µCT scaffold.

### 2.11 Statistical analysis

One‐way analysis of variance with Tukey post hoc test was conducted with GraphPad Prism 7.04 (GraphPad Software) for statistical analysis, where *p* ≤ .05 was considered as statistically significant. On the bar chart data are presented as mean ± the standard error of the mean.

## 3 RESULTS AND DISCUSSIONS

### 3.1 Cell viability and proliferation

**Figure 4:**
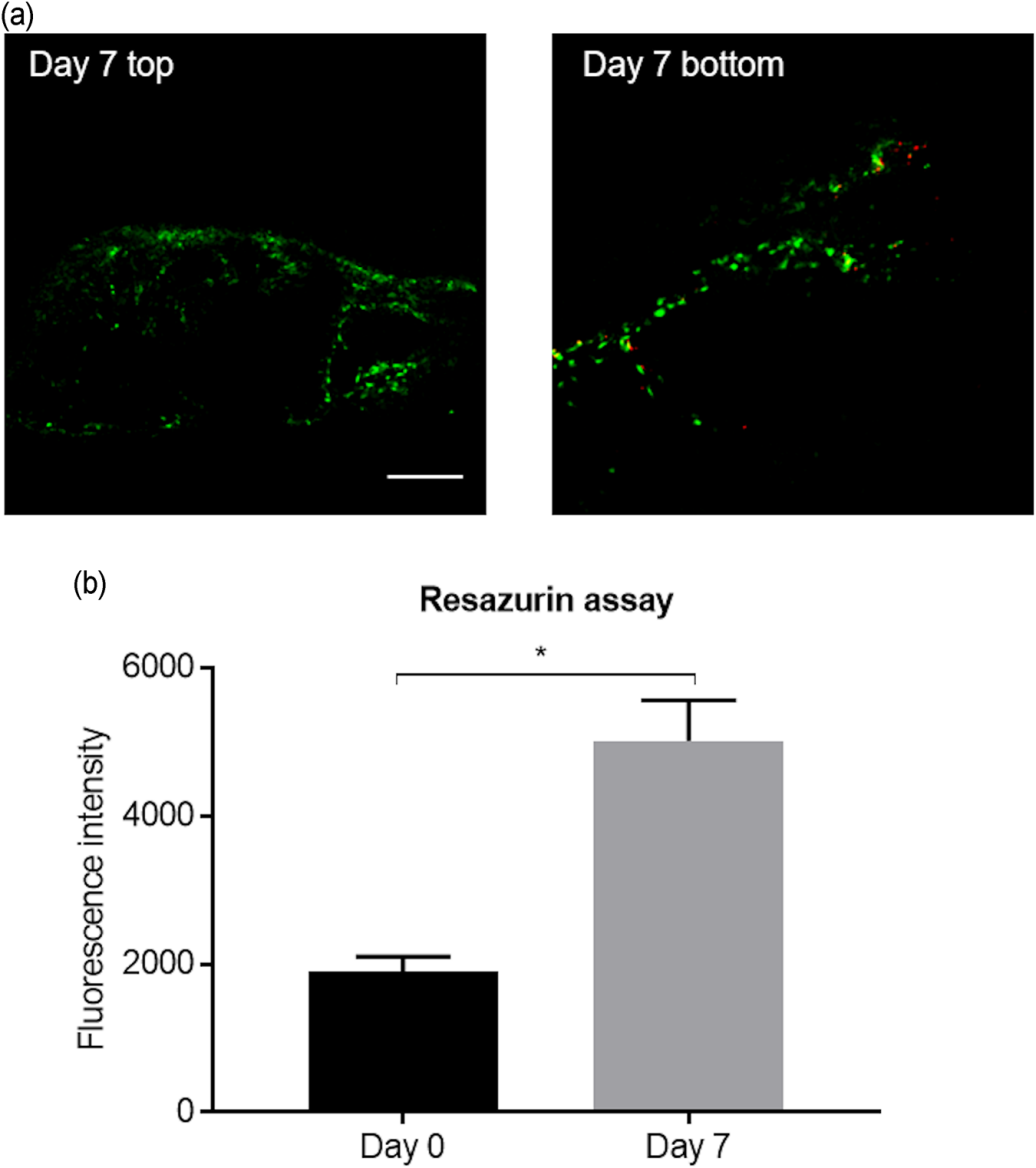
(a) Fluorescence micrographs of the top (mainly ATDC5 cells) and bottom (mainly MC3T3‐E1 cells) layer of the scaffold from the live/dead assay. Green cells were viable and red cells were dead. Scale bar is 250 µm. (b) Fluorescence intensity reading from resazurin assay on total cells in the scaffold at Day 0 and Day 7. * *p* ≤ .05 [Color figure can be viewed at wileyonlinelibrary.com]

Fluorescence micrographs from the Live/Dead assay revealed that most cells were viable at Day 7 on both top and bottom sections of the scaffold (Figure 4a). According to the resazurin assay (*n* = 3), the fluorescence intensity increased from approximately 1900 at Day 0 to 5000 at Day 7 (Figure 4b), which showed a significant increase in the metabolic activity and thus cell number during the 7‐day perfusion culture.

### 3.2 Cell distribution on the scaffold

**Figure 5:**
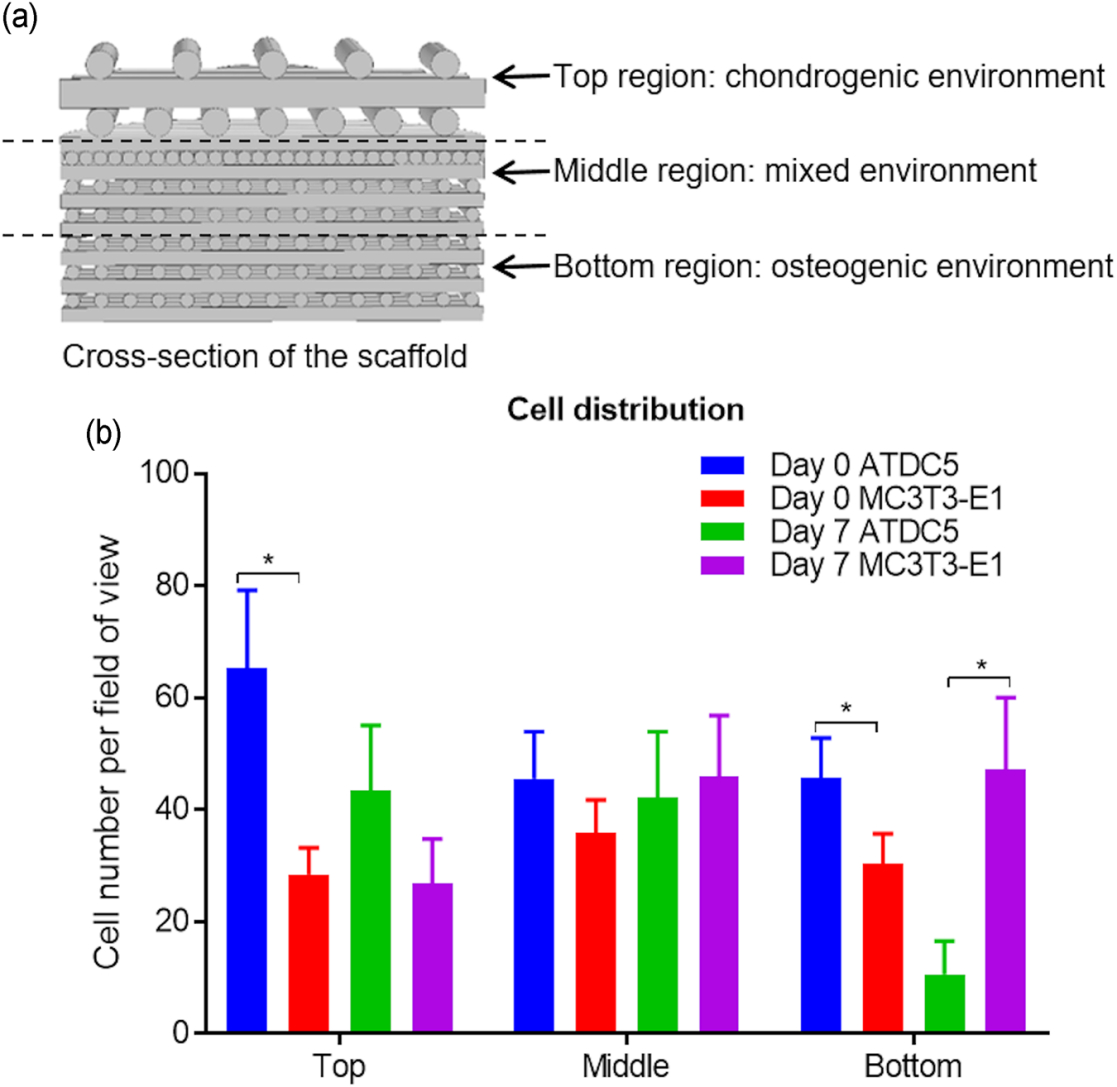
(a) Schematic of sections used for DiO and DiD cell tracking. (b) Cell number per micrograph at different sections of the scaffold for ATDC5 and MC3T3‐E1 cells at Day 0 and Day 7. *p ≤ .05 [Color figure can be viewed at wileyonlinelibrary.com]

For the DiO and DiD cell tracking analysis, the average number of ATDC5 and MC3T3‐E1 cells per fluorescence micrograph (*n* = 9) at the top, middle, and bottom section of the scaffold were calculated (Figure 5b). At Day 0, there were more ATDC5 than MC3T3‐E1 cells at the top section (66 to 32 cells; *p* = .05). Interestingly, more ATDC5 cells were also found at the bottom section (48 to 28 cells; *p* = .03). At Day 7, more ATDC5 than MC3T3‐E1 cells were found at the top section (43 to 27 cells; *p* = .26). Nevertheless, the ATDC5 cell number was significantly lower than MC3T3‐E1 at the bottom section 11–47 cells; *p* = .02).

To conclude, at Day 0, ATDC5 cells dominated both the top and the bottom sections whereas, at Day 7, ATDC5 cells dominated the top section and MC3T3‐E1 the bottom section. The result indicated that the cartilage and bone tissue‐specific environment created by the combination of the bioreactor and the bilayered scaffold had a positive effect on the cell distribution. The domination of ATDC5 cells at Day 0 was likely caused by the infiltration of cell suspension from the top to the bottom section because of the geometry of the scaffold (higher porosity and bigger pore size at the top section compared with bottom section). At Day 7, unfavorable microenvironment at the bottom section could lead to reduced cell attachment and number of ATDC5 cells.

### 3.3 Cell attachment to the scaffold

**Figure 6:**
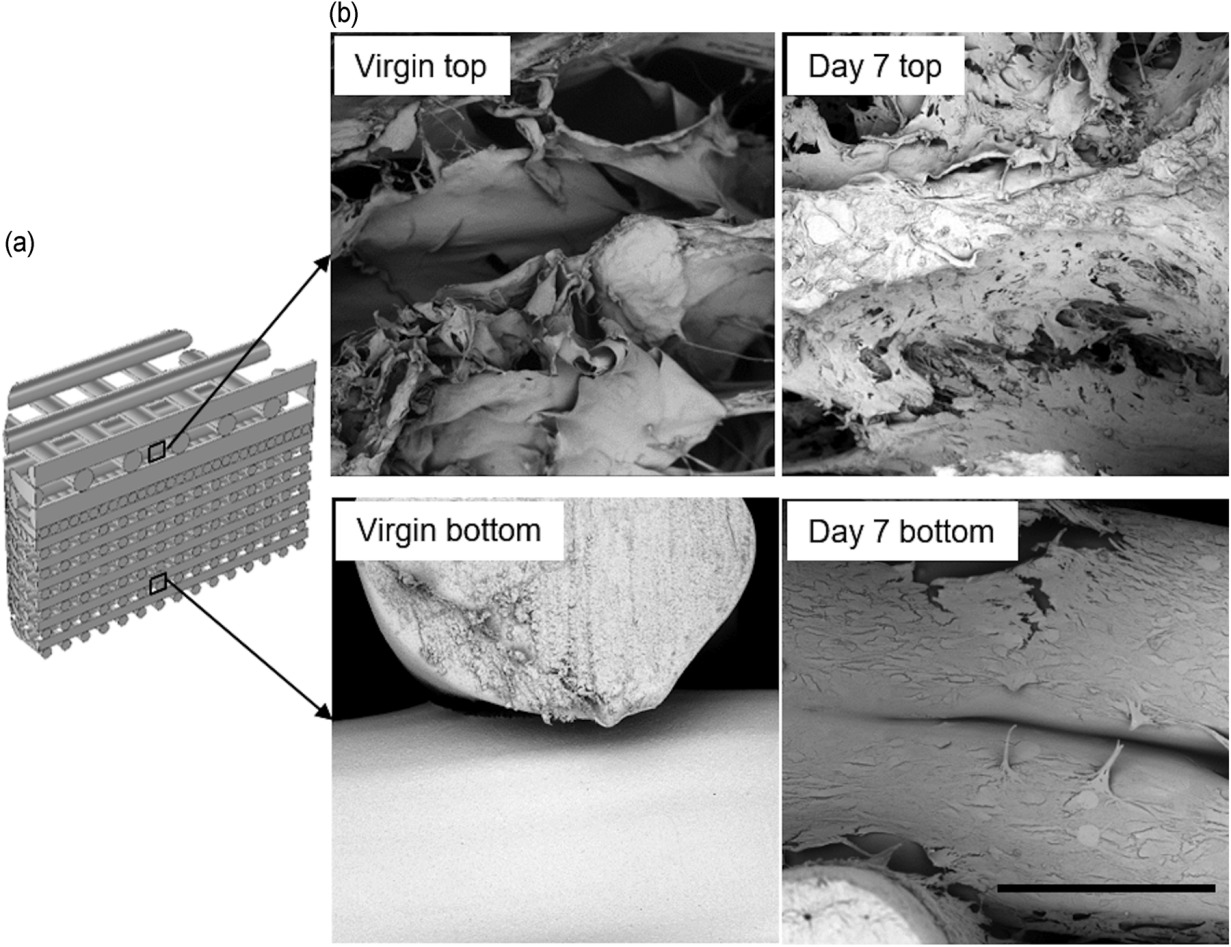
(a) Schematic of the location of the cells on the scaffold associated with the SEM micrographs. (b) Representative SEM micrographs of the top (mainly ATDC5 cells) and bottom (mainly MC3T3‐E1 cells) layer of the virgin scaffold and the cell‐seeded scaffold at Day 7. Scale bar = 300 μm. SEM, scanning electron micrograph

Figure 6 shows the representative SEM micrographs of the top (mainly collagen) and bottom (mainly PLA strut) sections of a virgin scaffold and a cell‐seeded scaffold at Day 7. SEM results revealed that cells were able to attach to both the collagen and PLA struts of the scaffold after the 7‐day perfusion. Furthermore, cells were also found bridging adjacent PLA struts.

### 3.4 Finite element analysis

**Figure 7:**
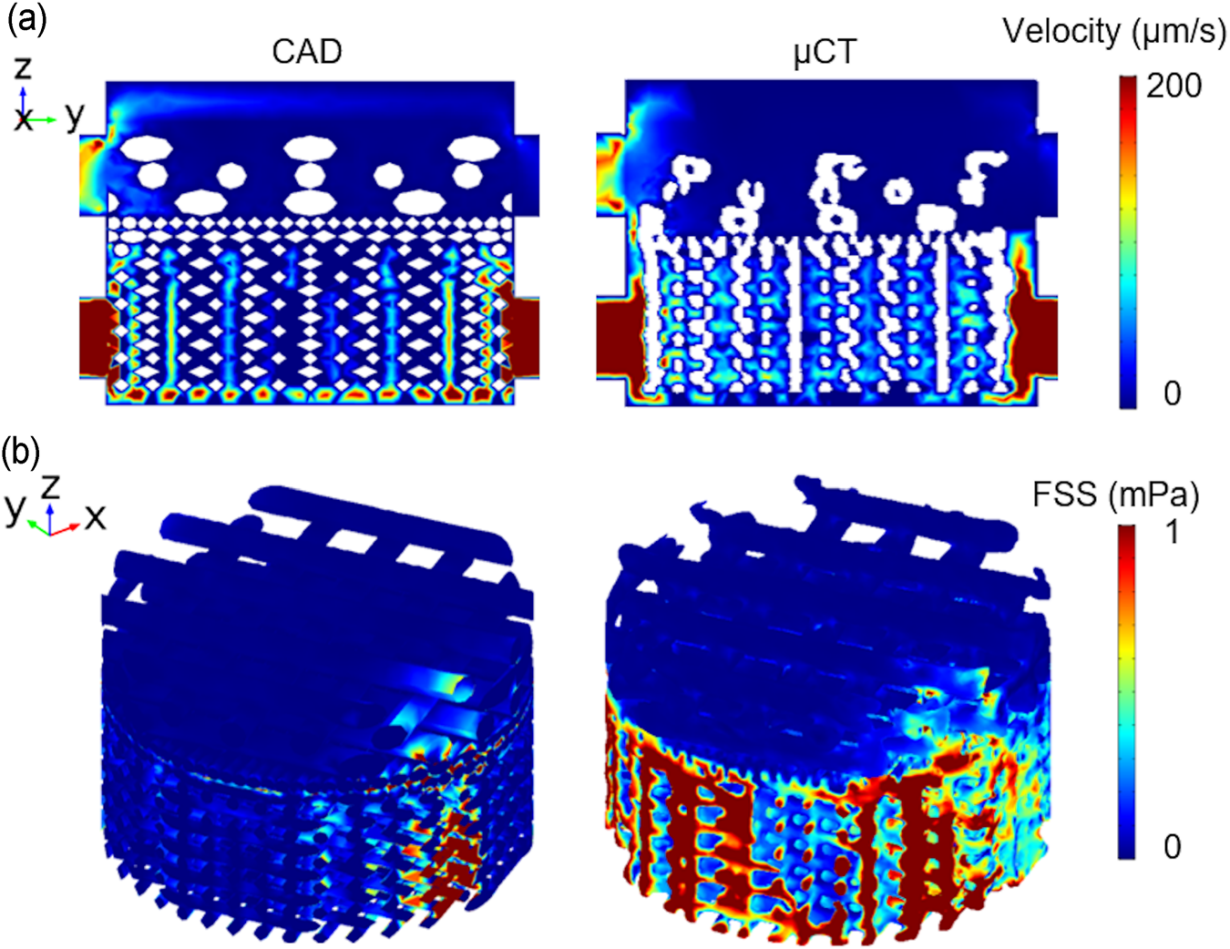
(a) Flow velocity distribution on the cross‐sectional slice for CAD and µCT model. (b) Fluid induced shear stress on the scaffold for the CAD and µCT model. CAD, computer aided design; μCT, microcomputed tomography [Color figure can be viewed at wileyonlinelibrary.com]

The FEA visualized the flow velocity inside the bioreactor chamber (Figure 7a). Noticeably, the flow velocity was highest near the inlets of the bottom section of the bioreactor, reaching 1320 μm/s for the model with CAD and 919 μm/s for the model with μCT image of actual manufactured scaffold (Table 1). In comparison, maximum flow velocity near the inlets of the top section of the bioreactor was 322 μm/s for CAD and 176 μm/s for µCT scaffold. Inside the scaffold, the flow velocity magnitude was much lower. The mean flow velocity in the respective chondral and osseous sections of the scaffold was 5.57 and 26.4 μm/s for the model with CAD, and 4.06 and 60.8 μm/s for the model with μCT image.

**Table 1:** Finite element analysis results of model with CAD and with µCT image

		CAD	µCT	Difference (%)
Mean V (µm/s)	Chondral	5.57	4.06	−27
	Osseous	26.4	60.8	+130
Max V (µm/s)	Chondral	322	176	−45
	Osseous	1320	919	−30
Mean FSS (mPa)	Chondral	0.0294	0.0296	+1
	Osseous	0.137	0.275	+101
Max FSS (mPa)	Chondral	12.4	3.35	−72
	Osseous	12.6	6.17	−51
Mean concentration (mM)	Chondral	0.960	0.966	+1
	Osseous	0.196	0.229	+17

Abbreviations: CAD, computer aided design; FSS, fluid‐induced shear stress; μCT, microcomputed tomography.

Compared with the inlet velocity (410 µm/s), the flow velocity is significantly lower because the cross‐sectional area of the chamber is much higher than the perfusion tubing. Flow velocity inside a 3D tissue‐engineered scaffold during the perfusion culture has been studied through computational modeling (McCoy, Jungreuthmayer, & O'Brien, 2012; Porter, Zauel, Stockman, Guldberg, & Fyhrie, 2005). Porter and co‐workers revealed that the flow velocity inside a decellularized trabecular bone (DTB) under perfusion culture ranged from 0 to 400 μm/s, which facilitates the nutrient and waste transport. It has been shown by McCoy et al that flow velocity over 235 μm/s was linked to increased detachment of bridging cells. In the current study, for both models, the velocity inside the scaffold is mainly under 200 µm/s, which provides sufficient mass exchange while minimizing cell detachment.

The FSS on the scaffold (Figure 7b) was revealed and quantified (Table 1). The maximum FSS was found at the areas close to the inlets and the outlets of the osseous layer, which was 12.6 and 6.17 for the respective CAD and μCT model. The FSS on the majority of scaffold surfaces was under 1 mPa, and the mean FSS in the respective chondral and osseous layers was 0.0294 and 0.137 mPa for the model with CAD and 0.0296 and 0.275 mPa for the model with μCT image.

From literature, the average fluid‐induced shear stress on the scaffold was reported to be in the range of 0.05–100 mPa depending on the scaffold geometry (e.g., porosity and pore size) and inlet velocity (Boschetti, Raimondi, Migliavacca, & Dubini, 2006; Maes et al., 2012; Porter et al., 2005; Zhang, Yuan, Lee, Jones, & Jones, 2014; Zhao, Vaughan, & Mcnamara, 2015; Zhao, Vaughan, & McNamara, 2016). It is worth noting that for the top (i.e., chondral) layer of the scaffold, the collagen was not captured by μCT due to very low attenuation under the X‐ray illumination. Instead, it was modeled as porous media for both CAD and μCT model. Thus, only the FSS on the PLA structure was considered.

**Figure 8:**
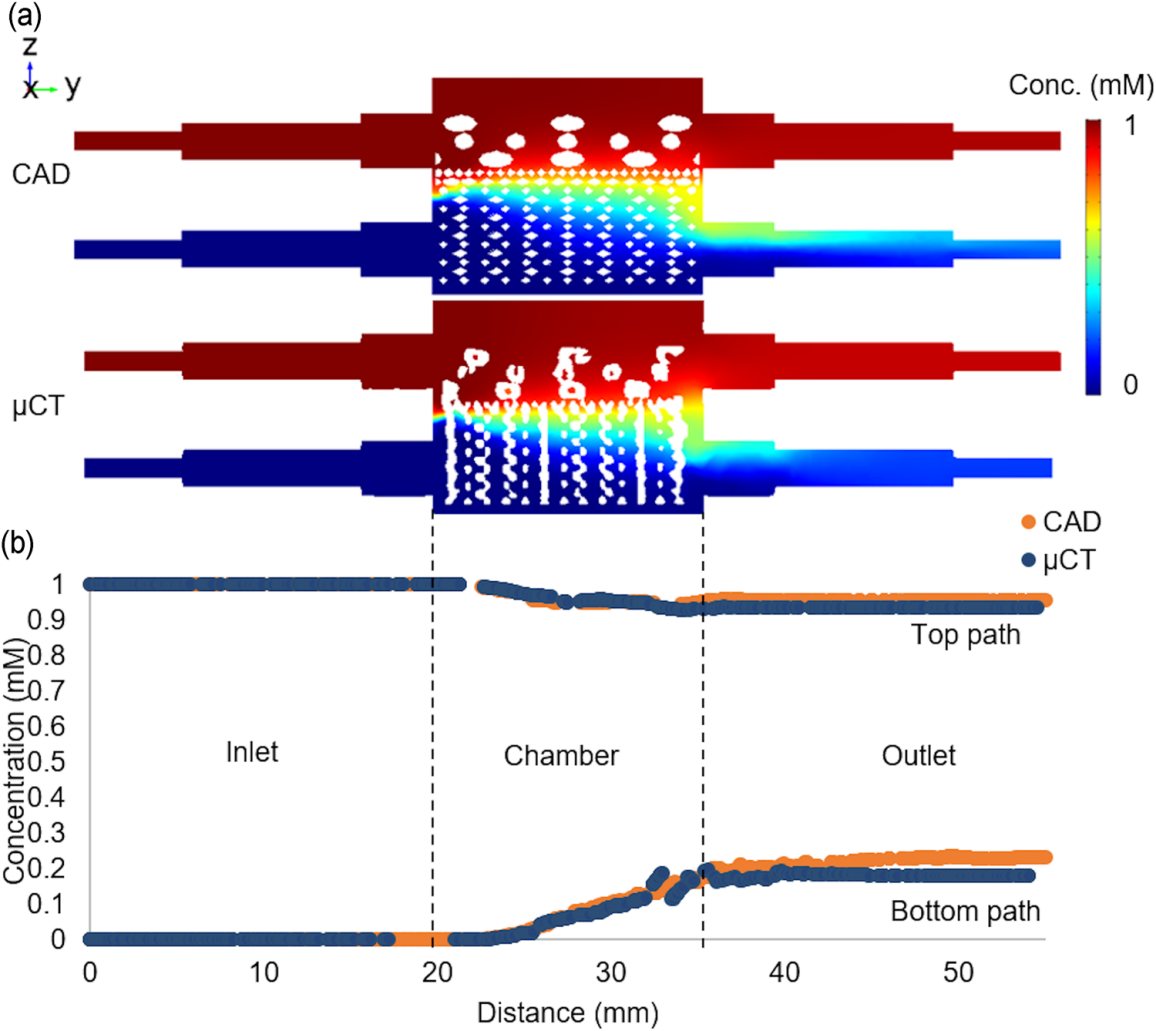
(a) Concentration on the cross‐sectional slice for CAD and µCT model. (b) Concentration profile from inlets to outlets for CAD and µCT model. CAD, computer aided design; μCT, microcomputed tomography [Color figure can be viewed at wileyonlinelibrary.com]

Figure 8a illustrates the mixing of the differentiation media on the cross‐sectional slice of the models. In the perfusion chamber, increased media mixing was observed at the region close to the outlets. Also, line profiles of the concentration from inlets to outlets were created in Figure 8b. At the outlets, there was a mixing of approximately 5% for chondrogenic medium and approximately 20% for osteogenic medium for both models. The mean media concentration at the respective chondral and osseous sections were 0.96 and 0.196 for the model with CAD and 0.966 and 0.229 for the model with μCT image.

Results showed that, in general, the different media were well contained in their respective sections. Various biochemical growth factors were often supplemented in the differentiation media to facilitate phenotype development (Vater, Kasten, & Stiehler, 2011). As bone and cartilage tissues require different growth factors to promote their respective phenotype development in vitro, a coculture system with minimal differentiation media mixing is desired for osteochondral tissue engineering (Alexander, Gottardi, Lin, Lozito, & Tuan, 2014; Vater et al., 2011).

The results with different scaffold orientation (Figure S1) showed that distributions of the flow velocity, FSS, and media mixing did not change significantly with scaffold rotation. Quantification data further confirmed the findings (Table S1).

Collagen and PLA are biodegradable materials with different degradation rate (García‐Gareta, Coathup, & Blunn, 2015); and the degradation process will likely cause a change in the microenvironment during the perfusion. However, the current FEM did not consider the degradation process. In the future, the FEM can be improved by incorporating the materials degradation profile through a time‐dependent study.

### 3.5 Discrepancy between the CAD and the actual manufactured scaffold

Comparing the model created with the CAD to that with the μCT image, the latter generally resulted in reduced flow velocity except for the mean velocity at the osseous section which saw a 130% increase (Table 1). For the FSS, increased mean magnitude but decreased maximum values were observed in the μCT model. For the media concentration, results showed a slight increase for the chondral layer and 17% increase for the osseous layer. The differences can be linked to the less homogeneous structure caused by common additive manufacturing methods including part accuracy, shrinkage, surface finish, and so on (Leong, Cheah, & Chua, 2003). Hendrikson et al. (2014) also reported that the CAD‐based FEM was not able to capture the distributions of shear strain and FSS seen in a μCT‐derived model, which was caused by more gradual geometry created with additive manufacturing (e.g., fewer sharp corners). They showed that both maximum and mean FSS magnitudes were higher in μCT data. Contrary to their results, higher mean FSS but lower maximum FSS magnitudes were observed in this study. The discrepancy can be caused by the different model geometry and perfusion boundary conditions used compared to a close fit regular scaffold in a cuboid perfusion chamber, a more complex model consisting of a realistic coculture perfusion chamber and a bilayered scaffold was used here. Thus, results from the μCT‐derived model were discussed below.

### 3.6 Microenvironment for osteochondral tissue engineering

For cartilage tissue engineering several studies reported that chondrocytes or cartilage progenitor cells showed enhanced chondrogenesis (i.e., upregulation of chondrogenic genes, increased collagen type II and GAG production) during in vitro 3D perfusion culture with up to 0.2 ml/min flow rate because of the increased mass transport when compared to static conditions (Goncalves et al., 2011; Alves da silva et al., 2011; Mahmoudifar & Doran, 2010; Pazzano et al., 2000). Pazzano et al. (2000) also showed that the flow perfusion was able to maintain the pH gradient throughout the scaffold leading to increased DNA content. However, other researchers found that the flow perfusion led to downregulation of SOX9, GAG, and collagen II expressions, indicating reduced chondrogenic and increased osteogenic differentiation (Guo et al., 2016; Kock, Malda, Dhert, Ito, & Gawlitta, 2014; Mizuno, Allemann, & Glowacki, 2001). The discrepancy can be caused by the higher flow rate used in those studies (0.33, 1, and1.22 ml/min), which led to increased FSS on the cells. Unlike for bone tissue, high FSS is not desired in cartilage regeneration. For instance, FSS up to approximately 0.1 mPa was used to maintain cartilage phenotype whereas 100 mPa was shown to reduce chondrogenesis (Gharravi et al., 2013; Guo et al., 2016). Furthermore, the culture media used in different studies were able to influence the cellular response to flow perfusion. Dahlin, Meretoja, Ni, Kasper, and Mikos (2014) found that without growth factor TGF‐β3, bovine articular chondrocytes showed more cartilage‐like phenotype under perfusion; however, with the addition of TGF‐β3, chondrogenic gene expression was suppressed by perfusion compared with the static control. Dahlin, Meretoja, Ni, Kasper, and Mikos (2013) also combined perfusion with the hypoxic environment, leading to improved chondrogenic differentiation.

For bone tissue engineering, it was reported that different shear stress led to different cellular behavior of MC3T3‐E1 cells seeded on a decellularized trabecular bone (DTB) after 7‐day perfusion culture (Cartmell, Porter, Garcia, & Guldberg, 2003; Porter et al., 2005). Shear stress of 0.05 mPa resulted in high‐cell viability and proliferation; 1 mPa led to high osteogenic gene expression, and 5 mPa resulted in significant cell death. Zhao, Chella, and Ma (2007) perfusion cultured human MSC on polyethylene terephthalate scaffolds for 20 days and found that appproximately 0.01 and 0.1 mPa shear stress led to increased proliferation and osteogenic expression, respectively. Similarly, whereas maintaining the mass transport (flow rate), Li, Tang, Lu, and Dai (2009) showed that the lower shear stress (5 mPa)‐induced higher cell proliferation and higher shear stress (> 10 mPa)‐induced upregulation of osteogenic gene of MSC on β‐TCP scaffolds at Day 28. In terms of maximum shear stress, Grayson et al. (2008) showed that 28 days perfusion cultured MSC had improved proliferation, osteogenic protein expression, mineral deposition, and cell distribution under 10 mPa compared with 2.6 mPa on a DTB scaffold. The discrepancy in magnitude of effective shear stress in the above publications could be caused by different scaffolds and cells, and methods used to estimate the shear stress magnitude.

**Figure 9:**
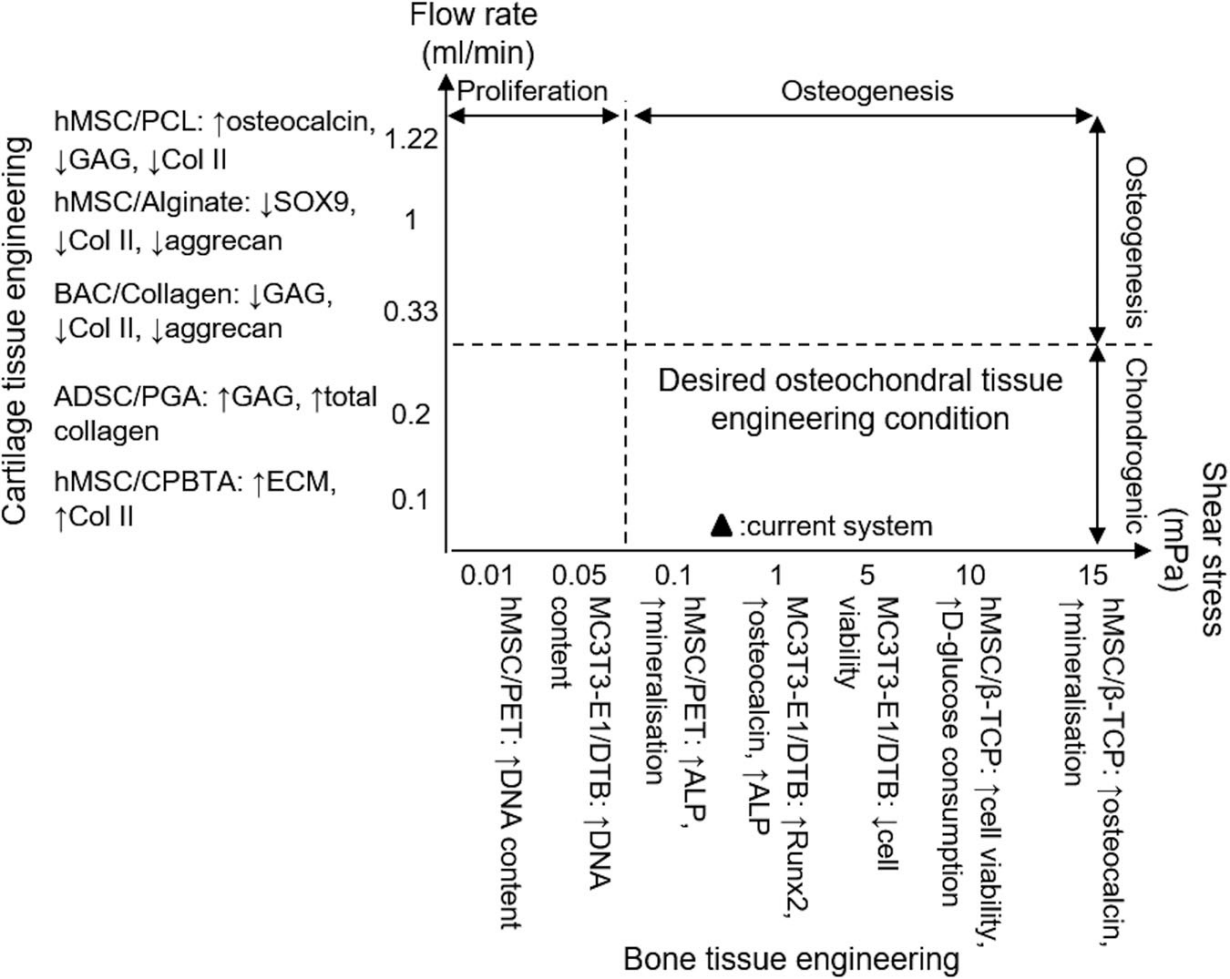
Bone and cartilage tissue engineering conditions for cells seeded on a 3D porous scaffold inside a perfusion bioreactor. For each study, cell type, scaffold material, and experiment outcome are presented as “Cell type/Scaffold material: experiment outcome”, followed by reference number. ADSC, adipose‐derived stem cell; ALP, alkaline phosphatase; BAC, articular chondrocyte; CPBTA, chitosan poly(butylene terephthalate adipate); ECM, extracellular matrix; PCL, polycaprolactone; PGA, polyglycolic acid; PLLA, poly l‐lactic acid

The effective regimes for osteochondral tissue engineering of 3D constructs are summarized according to the literature in Figure 9. The flow rate was chosen for cartilage tissue engineering because increased mass transport is the main purpose of perfusion here and data on FSS are very limited. The superficial velocity and the FSS highly depend on the chamber and scaffold geometry. Thus, ideally, the mean FSS needs to be calculated and controlled (< 0.1 mPa for chondrogenesis). The current system used a flow rate of 0.02 ml/min and induced mean FSS of approximately 0.03 and 0.28 mPa for the respective chondral and osseous layers, which lied in the effective osteochondral culture region. The flow velocity inside the scaffold supported the nutrient and waste transport whereas minimizing cell detachment. Also, it showed that the system was able to adequately maintain the respective osteogenic and chondrogenic medium, which facilitated the desired cell distribution. This system can be readily used as a preliminary and inexpensive platform for the efficacy test of medicinal products for osteoarthritis or drug delivery studies before conducting costly animal experiments at the early stage of development of new pharmaceutical products for osteochondral defects.

## 4 CONCLUSION

In conclusion, this study demonstrated that the current osteochondral culture system supports the coculture of ATDC5 and MC3T3‐E1 cells on a novel additive manufactured scaffold with regard to cell viability, proliferation, distribution”, and attachment. The microenvironment inside the bioreactor during the perfusion culture including flow velocity, fluid‐induced shear stress, and media mixing was studied using FEA. This system was shown to be viable in vitro osteochondral model due to its desirable microenvironment. It can be readily used as a platform for the cytotoxicity test or drug delivery study. For more clinically relevant applications like drug efficacy tests for osteoarthritis, the cell lines used can be easily replaced by primary cells or mesenchymal stem cells.

## Supporting information

Supplementary information

Supplementary information
