# Supplementary figures and images for: Osteochondral tissue coculture: An in vitro and in silico approach
Source: Biotechnol Bioeng. 2019 Jul 31;116(11):3112–23. doi: 10.1002/bit.27127 (PMC6790609; doi:10.1002/bit.27127)

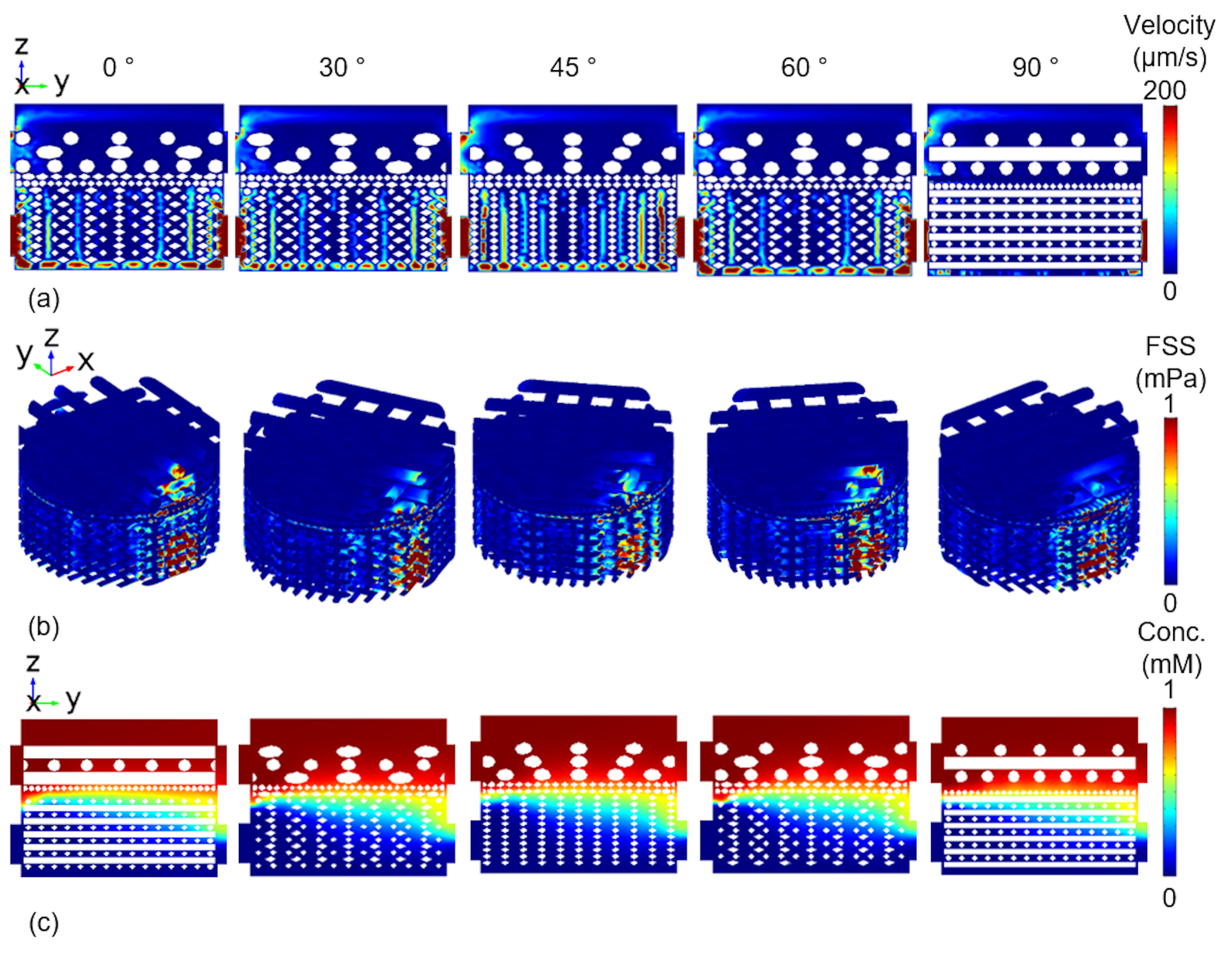

Supplement: Supplementary file 1 — Supplementary information [file BIT-116-3112-s001.tif]
