# Supplementary material for: Osteochondral tissue coculture: An in vitro and in silico approach
Source: Biotechnol Bioeng. 2019 Jul 31;116(11):3112–23. doi: 10.1002/bit.27127 (PMC6790609; doi:10.1002/bit.27127)
Supplement: Supplementary file 2 — Supplementary information [file BIT-116-3112-s002.docx]

Table S1. Finite element analysis results of model with CAD with different scaffold orientation.

| Scaffold rotation (°) | Mean V (µm/s) | | Mean FSS (mPa) | | Mean C (mM) | |
| --- | --- | --- | --- | --- | --- | --- |
|  | Chondral | Osseous | Chondral | Osseous | Chondral | Osseous |
| 0 | 5.58 | 27.4 | 0.0332 | 0.157 | 0.958 | 0.191 |
| 30 | 5.57 | 26.4 | 0.0294 | 0.137 | 0.960 | 0.196 |
| 45 | 5.62 | 25.8 | 0.0298 | 0.130 | 0.958 | 0.185 |
| 60 | 5.06 | 27.5 | 0.0295 | 0.138 | 0.953 | 0.184 |
| 90 | 5.90 | 27.8 | 0.0297 | 0.142 | 0.963 | 0.204 |
| Average | 5.55 | 27.0 | 0.030 | 0.141 | 0.958 | 0.192 |
